# Effect of Edge-to-Edge Mitral Valve Repair on Chordal Strain: Fluid-Structure Interaction Simulations

**DOI:** 10.3390/biology9070173

**Published:** 2020-07-18

**Authors:** Milan Toma, Daniel R. Einstein, Keshav Kohli, Sheridan L. Caroll, Charles H. Bloodworth, Richard P. Cochran, Karyn S. Kunzelman, Ajit P. Yoganathan

**Affiliations:** 1 Department of Osteopathic Manipulative Medicine, College of Osteopathic Medicine, New York Institute of Technology, Old Westbury Campus, Northern Boulevard, Old Westbury, NY 11568-8000, USA; 2 Wallace H. Coulter Department of Biomedical Engineering, Georgia Institute of Technology and Emory University, Technology Enterprise Park, 387 Technology Circle, Atlanta, GA 30313-2412, USA; kkohli@gatech.edu (K.K.); scarroll9@gatech.edu (S.L.C.); cbloodworth3@gatech.edu (C.H.B.IV); ajit.yoganathan@bme.gatech.edu (A.P.Y.); 3 Department of Mechanical Engineering, St. Martin’s University, 5000 Abbey Way SE, Lacey, WA 98503, USA; DEinstein@stmartin.edu; 4 Department of Mechanical Engineering, University of Maine, 219 Boardman Hall, Orono, ME 04469-5711, USA; cochranpat@superiorsurgicalsolutions.com (R.P.C.); kunzelka@superiorsurgicalsolutions.com (K.S.K.)

**Keywords:** mitral valve, edge-to-edge repair, chordae tendineae, comprehensive model, fluid-structure interaction, computer simulation, chordal strain

## Abstract

Edge-to-edge repair for mitral valve regurgitation is being increasingly performed in high-surgical risk patients using minimally invasive mitral clipping devices. Known procedural complications include chordal rupture and mitral leaflet perforation. Hence, it is important to quantitatively evaluate the effect of edge-to-edge repair on chordal integrity. in this study, we employ a computational mitral valve model to simulate functional mitral regurgitation (FMR) by creating papillary muscle displacement. Edge-to-edge repair is then modeled by simulated coaptation of the mid portion of the mitral leaflets. in the setting of simulated FMR, edge-to-edge repair was shown to sustain low regurgitant orifice area, until a two fold increase in the inter-papillary muscle distance as compared to the normal mitral valve. Strain in the chordae was evaluated near the papillary muscles and the leaflets. Following edge-to-edge repair, strain near the papillary muscles did not significantly change relative to the unrepaired valve, while strain near the leaflets increased significantly relative to the unrepaired valve. These data demonstrate the potential for computational simulations to aid in the pre-procedural evaluation of possible complications such as chordal rupture and leaflet perforation following percutaneous edge-to-edge repair.

## 1. Introduction

Mitral valve regurgitation (MR) is a highly prevalent disorder which is associated with significant morbidity and mortality if left untreated. However, 49% of patients are turned down for conventional treatment of MR with surgical mitral valve (MV) repair or replacement due to high surgical risk [1]. Minimally invasive MV repair and replacement devices are therefore being evaluated as alternative strategies for alleviating symptomatic MR in patients ineligible for surgical treatment.

**Figure 1:**
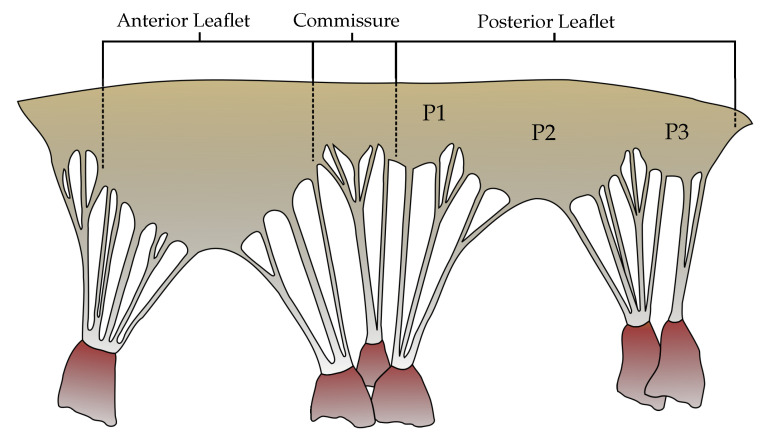
The geometry of unfolded MV; the papillary muscles attach via chordae tendineae to the anterior, posterior and commissural leaflets [2].

The left atrium receives oxygen-rich blood from the lungs and pumps it to the left ventricle through MV. The MV is therefore responsible for maintaining forward flow of blood from the lungs to the rest of the body. The mitral apparatus is composed of the left atrial wall, the annulus, the leaflets, the chordae tendineae, the papillary muscles, and the left ventricular myocardial wall. The MV alone is a complex structure which is composed of (1) the anterior and posterior mitral leaflets, (2) a sub-valvular apparatus consisting of chordae tendineae and papillary muscles (PMs). The anterior leaflet is comprised of one contiguous scallop and the posterior leaflet is comprised of the smaller P1, P2, and P3 scallops (Figure 1). MV closure during systole depends on a coordinated force balance across both the valvular and sub-valvular structures. Accordingly, loss of function of the sub-valvular apparatus (e.g., due to chordal rupture) disrupts the link between the MV and the left ventricle (LV), causing MR. More detailed specification can be found in [2].

There are two primary etiologies for MR: degenerative MR (DMR) and ischemic MR (e.g., chordal rupture caused by myocardial infarction) [3]. DMR occurs from disease of the MV tissue, such as fibroelastic deficiency or Barlow’s disease. Functional MR (FMR) is secondary etiology primarily due to LV dilation causing PM displacement, leaflet tethering, and annular dilation [4]. LV dilation can increase the distance between PMs causing FMR and leaflet tethering. However, it is believed that the sole distance between the PMs is not a direct determinant of FMR [5,6].

Edge-to-edge (E-to-E) repair is a surgical MV repair technique first introduced in the 1990s in which the anterior and posterior MV leaflets are sutured together at the precise location of the regurgitant jet to improve leaflet coaptation, thus creating a double orifice MV and reducing the degree of MR [7]. The inherent simplicity of this surgical technique made it ideal for direct translation into a catheter-based approach. As such, several transcatheter devices are undergoing development and clinical evaluation for the treatment of MR using the E-to-E repair technique.

MitraClip (Abbott Vascular, Abbott Park, IL, USA) is currently the only FDA-approved catheter-based MV repair system for the treatment of DMR, and has been performed in over 40,000 patients worldwide [8]. The device consists of two mechanical arms designed to grasp the anterior and posterior MV leaflets, simulating E-to-E surgical repair. Additionally, the first-in-man experience with the PASCAL MV repair system (Edwards Lifesciences, Irvine, CA, USA) has recently been reported and showed a consistent reduction in MR severity following percutaneous E-to-E repair [9]. Moreover, recent clinical data suggests that transcatheter E-to-E repair is an effective strategy for treating MR and is well tolerated in select high risk patients [10,11].

Despite the growing clinical experience, there are still some key challenges associated with transcatheter E-to-E repair. in particular, placement of a mitral clip device may increase the risk for rupturing the mitral leaflets or chordal apparatus. This risk is elevated in the presence of mitral leaflet tethering [12]. The intra-chordal strain can be assessed pre-operatively using computational modeling techniques to predict the patient-specific risk for causing leaflet or chordal rupture post-clip placement. in order to perform these analyses, a granular understanding of MV mechanics must first be acquired.

To understand the mechanics governing MV function, finite element analysis was introduced decades ago for normal [13], diseased [14,15,16], and surgically repaired states [17,18,19,20]. More recently, we and others have utilized fluid-structure interaction (FSI) analysis to understand the complex dynamics of MV function [21,22,23,24,25,26]. Most of these models utilized MV geometries obtained by either (1) parametric modeling, (2) image reconstruction of markers placed on the valvular apparatus, or (3) excised valve specimens. More recently, MV modeling strategies have been transitioning to morphologically realistic patient-specific MV modeling utilizing real-time 3D imaging data [27,28,29]. However, one of the obstacles limiting patient-specific modeling has been the inability to accurately represent the complex chordal structure of the native valve.

To increase computational efficiency, most current models of the MV use simplified element types to represent chordae tendineae and leaflet geometry. Models commonly take advantage of the chordal cable-type structure to justify the use of rod elements and the thinness of the leaflets to justify the use of shell elements [24,30,31,32,33,34,35,36]. Some models do represent the leaflets with 3D elements (e.g., tetrahedral) but often only utilize a single layer to reduce computational time. Furthermore, the detailed 3-D branching nature of the chordae has not been well-represented in current computational models of the MV. Geometric simplifications of existing models may limit the accuracy of the computational results. We propose that improved anatomic model accuracy is necessary for a thorough investigation of valvular dynamics.

We have recently developed and validated an ex vivo MV imaging methodology and associated computational platform, wherein the subject-specific MV geometry can be modeled with an unprecedented level of leaflet and chordal detail. For the first time, secondary and tertiary chordae are incorporated into FSI simulations of MV closure using a detailed finite element model [25,37,38,39]. We have shown that the use of FSI analysis is necessary when investigating the effect of a repair technique on the chordae tendineae [37]. The model has been validated from multiple perspectives including comparing PM forces and leaflet closure with experimental and in vivo data [25,40]. The fully validated model was then used to assess one of the chordae-related pathologies, namely chordal rupture [38].

While computational modeling of transcatheter E-to-E repair has been performed on simplified, symmetric valve representations where the chordae are either modeled as spring elements or excluded altogether [41,42,43], E-to-E repair has not been simulated using an anatomically accurate representation of the MV apparatus. Importantly, native mitral chordal tree topology has a specific structure, which distributes loads along the leaflets in distinct patterns [44]. Therefore, for accurate simulation results, it is critical to simulate E-to-E repair using the native mitral chordal topology as compared with a simplified chordal topology.

A computational model by Votta et al. also simulated annular dilation with E-to-E repair and noted an increase in axial stress in the chordae near the E-to-E repair [41]. Our study extends the findings presented in Votta’s paper by examining the load distributions on subject-specific mitral chordal tree and valve using FSI analysis, in contrast to the idealized valve and chordal structure using structural analysis (as in previous studies). To our knowledge, the present study represents the first ever report of FSI analysis used for pre-procedural simulation of MV repair mechanics and evaluation of subject-specific risk or chordal or leaflet rupture after transcatheter E-to-E repair using anatomically accurate MV models.

## 2. Materials and Methods

### 2.1. Model Acquisition

**Figure 2:**
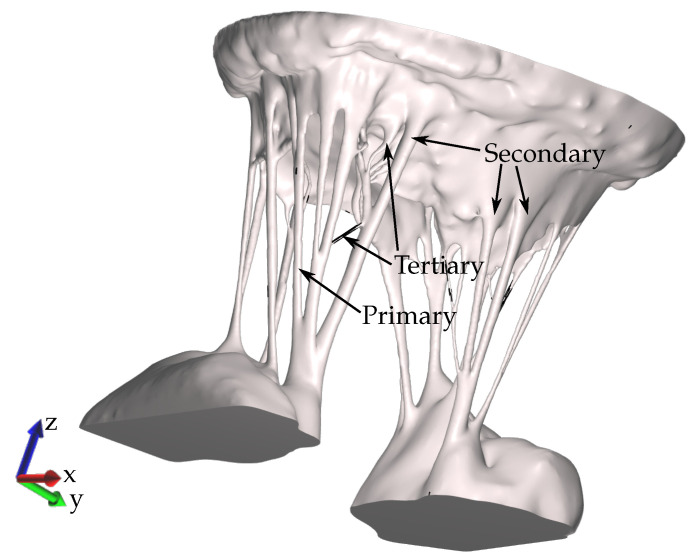
The detailed MV model extracted from DICOM μCT images with all chords preserved.

A novel technique for treating MV specimens in preparation for micro computed tomography (μCT) scanning was used to obtain an MV image significantly more detailed in chordal structure, accurate in leaflet shape, and close to its physiological diastolic geometry than ever before achieved [40,45]. The resulting geometry is shown in Figure 2.

### 2.2. Fluid-Structure Interaction Analysis

Fluid motion and boundary interaction was solved with the IMPETUS Afea SPH Solver^®^ (IMPETUS Afea AS, Norway), while large deformation in the solid mitral valve was simultaneously solved with the IMPETUS Afea Solver^®^. Both the solvers use a commodity GPU for parallel processing. All solid elements were fully integrated removing the possibility of hourglass modes and element inversion that plagues the classic under-integrated elements. Standard numerical methods for solving FSI problems with high number of degrees of freedom are more challenging to parallelize [46].

Both fluid and solid domains and their interaction were solved with an explicit integration scheme. All simulations were solved on a standard workstation. Parallel acceleration was achieved with a Tesla K40 GPU with 12 GB of Graphic DDR memory and 2880 CUDA Cores. To confirm that convergence was reached, h-refinement of the finite element mesh was performed and the solution was found to yield same results.

**Figure 3:**
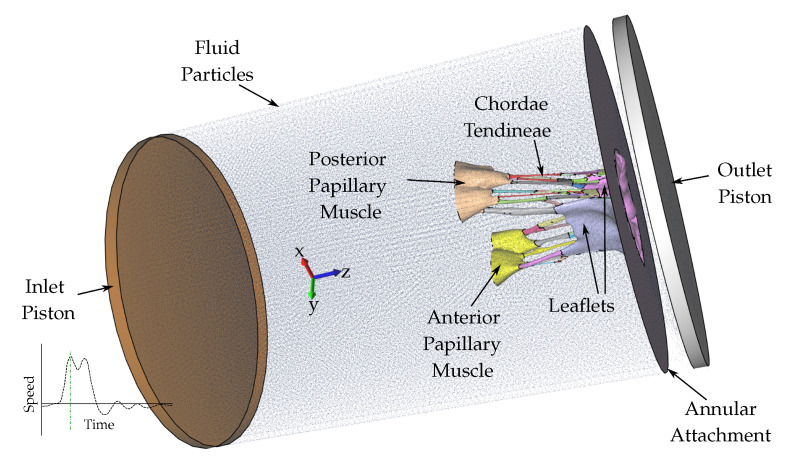
The fluid particles are confined in a pipe-like rigid structure surrounding the MV model and prescribed velocity boundary conditions are applied to the open ends via the use of moving pistons in z-direction.

The fluid particles were confined in a pipe-like rigid structure surrounding the model, and two pistons moved within the pipe at a prescribed velocity (Figure 3). The nodes at the bottom of the PMs were fixed in all three directions, as well as the nodes on the annular attachment. The MV model consisted of 92,640 tetrahedral elements, and 476,199 fluid particles were used to represent the fluid. To confirm that convergence was reached, refinement and coarsening of the finite element mesh (h-refinement) were performed. After testing meshes of three different resolutions (92,640 elements, ±20%), all solutions were found to converge to within 5% of each other.

The constitutive model for the material properties utilized a three-dimensional splay invariant, based on an approximation of a three-dimensional Gaussian distribution of fibers [47]. The material parameters and more detailed description of the material model is presented in the previous papers utilizing this model [25,37].

### 2.3. Validation

The results of the FSI simulations using the above described model and its material properties have been validated against experimental data in two ways. Firstly, the direction and magnitudes of PM forces were compared with experimental measurements [25] and, secondly, the coaptation line between the anterior and posterior leaflets at closure has been compared with the μCT images [40].

### 2.4. Papillary Muscles Displacements and Edge-to-Edge Repair

Following reconstruction of a healthy mitral valve, the IMPETUS Afea Solver^®^ has been used to simulate the functional MV regurgitation by incrementally displacing the PMs. To simulate the E-to-E repair, again the IMPETUS Afea Solver^®^ was used to pull the leaflets together using spring elements. Subsequently, an FSI analysis using the IMPETUS Afea SPH Solver^®^ was performed for each increment with differing distance between the PMs with and without E-to-E repair.

### 2.5. Regurgitant Orifice Area Measurements

To define the change in ROA, healthy MV closure was first simulated. Nodes along the mitral leaflet coaptation line were selected forming a coaptation line boundary. The ROA was estimated by calculating the total (3D) area bounded by these same nodes in subsequent simulations.

### 2.6. Strain Assessment

As observed in the results, the highest strain values on the chordae tendineae were found close to the PMs and leaflets. For comparison, after each FSI simulation the strain values were recorded on each chorda close to both the leaflets and PMs. The values near the leaflets were then averaged to compare the resulting value with the average one near the PMs. All measurements were performed for valves with and without E-to-E repair with varying distance between PMs (resulting in varying ROA). It can be seen that the difference in ROA becomes significant when the distance between the PMs is twice the distance in the healthy valve model without PM dilation.

## 3. Results

**Figure 4:**
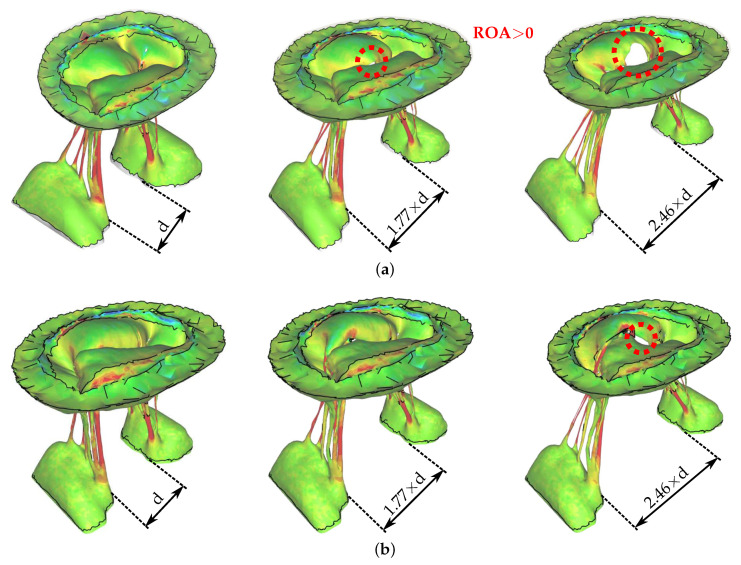
The effect of edge-to-edge (E-to-E) repair on the regurgitant orifice area (ROA) that resulted from dilated papillary muscles. (**a**) Without E-to-E repair. (**b**) With E-to-E repair.

**Figure 5:**
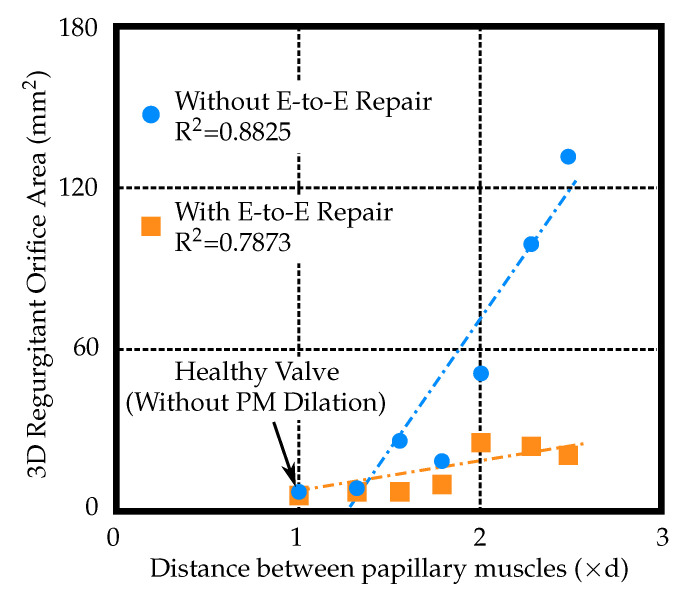
Regurgitant orifice area versus papillary muscles (PM) displacement (where ‘d’ is the distance between papillary muscles of the healthy valve model, i.e., without PM dilation).

The effect of E-to-E repair on the regurgitant orifice area (ROA) that resulted from dilated PMs can be seen in Figure 4. The first row depicts the valve without the E-to-E repair with three different distances between the PMs. Large ROA (≈130 mm${}^{2}$) can be observed in the case with the most dilated PMs. The corresponding figures in the second row show the effect of E-to-E repair on the ROA shown in the first row. A small non-zero ROA (≈20 mm${}^{2}$) can still be observed in the case with the most dilated PMs even after E-to-E repair. However, ROA of 20 mm${}^{2}$ is a significant improvement compared to 130 mm${}^{2}$ without the E-to-E repair. All ROA measurements versus varying PM displacements are shown in Figure 5.

**Figure 6:**
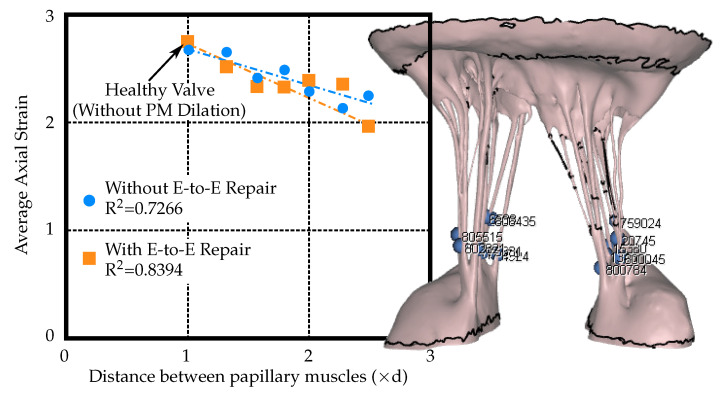
Average strain values in the chordae near the papillary muscles (PM) versus PM displacement (where ‘d’ is the distance between papillary muscles of the healthy valve model, i.e., without PM dilation).

**Figure 7:**
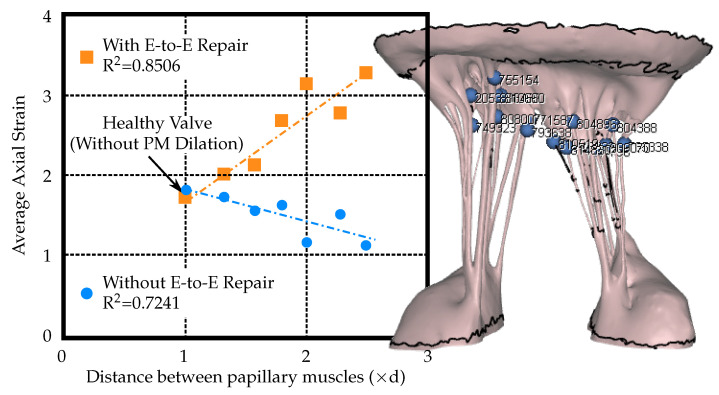
Average strain values in the chordae near the mitral valve leaflets versus papillary muscles (PM) displacement (where ‘d’ is the distance between papillary muscles of the healthy valve model, i.e., without PM dilation).

The axial strain in the points evaluated close to the PMs showed little variation between the percutaneous E-to-E repaired and unrepaired model, see Figure 6. Both models showed a trend of decreasing strain with increasing PM displacement. The axial strain in the points on the chordae towards the leaflets increased in the percutaneous E-to-E repaired model and decreased in the unrepaired model (Figure 7). Corresponding points where the axial strain values were measured are depicted in Figure 6 and Figure 7. The measurement points where chosen at the locations where chordae attached to the leaflets and PMs.

## 4. Discussion

In this study, we employed a form of computational modeling known as FSI analysis to model the subject-specific device-tissue interaction following transcatheter E-to-E repair. Our study focused on evaluating the efficacy of E-to-E repair in subject-specific models of FMR. We simulated varying levels of PM displacement, and measured the resultant ROA following E-to-E repair. We utilized a unique computational model that maintained the subject-specific 3D chordal structures, retained mesh element integrity, and used FSI analysis to simulate the MV closure. Hence, we have demonstrated that computational modeling can identify potential areas of failure in E-to-E repair based on the underlying MV pathology.

Our simulations of FMR appropriately increased the ROA, suggesting that the alterations to our model successfully simulated functional MV pathology. When E-to-E repair was applied, the ROA values reduced significantly compared to the values measured on the unrepaired model. As the inter-papillary muscle distance increased, the ROA increased regardless of whether the E-to-E repair technique was applied. However, this increase in ROA was of significantly smaller magnitude as compared to the unrepaired FMR model. The E-to-E repair is commonly performed with an undersized annuloplasty which stabilizes the repair and reduces stress on the E-to-E suture. Without annuloplasty, the freedom from re-operation or recurrent MR is lower and has been reported to reduce from 95% to 79% [7]. Depending on the severity of MV regurgitation, multiple percutaneous E-to-E repairs have been used on one valve.

The strain values of select chordae near the leaflets and papillary muscles were evaluated as the distance between the PMs was increased. The strain in the chordae near the leaflets was observed to nearly double when the E-to-E repair was applied. However, the strain in the chordae near the papillary muscles remained consistent. Without E-to-E repair, the strain values decreased as PM distance increased. This is an expected finding, since a larger ROA corresponds to more regurgitant flow and therefore decreased forces imparted on the MV leaflets and chordae by the fluid.

We also found that the areas near the leaflets can have a higher potential of chordal rupture due to cross-sectional area differences and/or leaflet proximity. Additionally, when the fold change in PM distance was larger than reported, a chordae also ruptured in the model. Therefore, this computational study corroborates the clinical findings that clipping the mitral leaflets in patients with severe PM displacement and leaflet tethering can lead to chordal rupture. in particular, the additional strain placed on the upper chordae by the E-to-E repair can significantly impact future MV integrity.

By all means, limitations of the methods and models used need to be listed. The model is based on a single patient-specific geometry. Hence, this is a single case study. More models need to be processed for statistically significant results. The MV used to create the model was excised from a healthy porcine heart. Therefore, while the geometry is patient-specific, the material properties are based on previously published measurements of a healthy porcine MV. For that reason, MV material properties used here are assumed to be well representative as well. In one of our studies, we showed that even when two different species are used, the results obtained are comparable [25]. Currently, this model is acquired with high difficulty, i.e., the MV must be excised, placed in a chamber, stabilized, and imaged using μCT [40]. Next, resulting CT images are used to create the 3D model. The 3D model is then processed and boundary conditions are set. Each simulation takes up to 24 h to complete. As the model acquisition and subsequent processing of the model are both laborious tasks, currently this study is only meant to provide insight into the processes governing the percutaneous E-to-E repair. Before it can be used in clinical practice, further development is necessary. This study may generate hypothesis on how chordal strain is important to determine post-operative complications, but it needs to be supported by clinical evidence.

## 5. Conclusions

Percutaneous E-to-E repair with mitral clipping devices is often the only option for high risk patients with severe, symptomatic MR. The E-to-E repair technique is currently being used effectively to treat both FMR and DMR patient populations. in this study, we simulated the FMR etiology by creating PM displacement in a subject-specific MV model. However, the mitral annular size remained unchanged in our FMR model. it is expected that with left ventricular dilation in the setting of FMR, the mitral annulus would increase in size as well. However, this annular dilation would yield even larger chordal strain values after E-to-E repair than what were observed in this study. Regardless, future investigations can assess the impact of annular dilation on chordal and leaflet rupture following E-to-E repair.

Despite the limited risks of leaflet and chordal rupture, percutaneous E-to-E repair remains a very effective method to reduce MR in high risk patients with no alternative surgical options. Recently, computational modeling of the MV has been advancing towards patient specific simulations. Altering our existing model with patient specific MV parameters is the next step towards personalizing these simulations. Herein, we have demonstrated that modifications to a normal MV model can successfully produce FMR in silico. We have also shown that modeling E-to-E repair can effectively reduce the simulated MR. in total, these data demonstrate the potential for computational simulations to aid in the pre-procedural evaluation of possible complications such as chordal rupture and leaflet perforation following percutaneous E-to-E repair.

## Abbreviations

The following abbreviations are used in this manuscript:

FMR	Functional Mitral Regurgitation
MR	Mitral Regurgitation
MV	Mitral Valve
PMs	Pupillary Muscles
LV	Left Ventricle
DMR	Degenerative Mitral Regurgitation
FMR	Functional Mitral Regurgitation
E-to-E	Edge-to-Edge
FDA	Food & Drug Administration
FSI	Fluid-Structure Interaction
ROA	Regurgitant Orifice Area
μCT	Micro Computed Tomography
DICOM	Digital Imaging and Communications in Medicine
SPH	Smoothed Particle Hydrodynamics
